# Size of portally deprived liver lobe after portal vein ligation and additional partial hepatectomy: Result of balancing proliferation and apoptosis

**DOI:** 10.1038/s41598-020-60310-0

**Published:** 2020-03-17

**Authors:** Weiwei Wei, Chuanfeng Hua, Tianjiao Zhang, Olaf Dirsch, Felix Gremse, André Homeyer, Utz Settmacher, Uta Dahmen

**Affiliations:** 1 Department of General, Visceral and Vascular Surgery, Jena University Hospital, Jena, Germany; 2 Department of Radiotherapy and Radiooncology, Jena University Hospital, Jena, Germany; 3 Institute of Pathology, Klinikum Chemnitz gGmbH, Chemnitz, Germany; 4 Department of Experimental Molecular Imaging, RWTH Aachen University, Aachen, Germany; 5 Fraunhofer Institute for Medical Image Computing MEVIS, Bremen, Germany

**Keywords:** Hepatic portal vein, Hepatocytes

## Abstract

The liver has the ability to maintain its total size by adjusting the size of the individual liver lobes differently in response to regeneration- and atrophy-stimuli. Portal vein ligation (PVL) drives the ligated lobe to undergo atrophy whereas partial hepatectomy (PHx) drives the total remnant liver to regenerate. We hypothesize that the size of the PVL-lobe is dependent on the balance between the extent of PVL and the extent of PHx inducing a complex interplay between hepatocyte proliferation, apoptosis and autophagy. Lewis-rats were subjected to either 20%PVL + 70%PHx or 70%PVL + 20%PHx. Control groups consisted of 20%PVL and 70%PVL. Liver lobe weight, BrdU-proliferation-index, proliferating-cell-nuclear-antigen-mRNA-expression level, apoptotic density and autophagy-related-proteins were investigated. The PVL-liver lobe adjusted its weight differently, increasing by 40% after 20%PVL + 70%PHx, but decreasing by 25% after 70%PVL + 20%PHx. Additional resection induced a low, but substantial size-dependent hepatocyte proliferation rate (maximal 6.3% and 3.6% vs. 0.3% and significantly suppressed apoptotic density in the deportalized-liver-lobe (3 and 14 cells/mm^2^ comparing with above 26 cells/mm^2^, *p* < 0.01). Autophagy was more activated in PVL-liver lobe after simultaneous PHx than after PVL only. In summary, atrophy of the PVL-liver lobe after simultaneous PHx was counteracted by promoting hepatocyte proliferation, inducing autophagy and suppressing apoptosis in a PHx-extent-dependent manner.

## Introduction

The liver has the tendency to maintain its size in case of partial hepatectomy or other liver injuries^1^. This is achieved by its unique ability to compensate the loss of liver mass by regeneration. However, the liver can not only regenerate but also undergo atrophy when deprived from portal blood. In this case, the process of intrahepatic size regulation starts: Atrophy of the portal vein ligated (PVL) lobes drives the non-ligated lobes to initiate hepatocyte proliferation and thus to undergo hypertrophy^2^. In contrast, partial hepatectomy (PHx) drives the total remant liver to undergo hypertrophy. In other words the liver has the remarkable potential to maintain its total volume by adjusting the size of the single lobe differently in response to extrahepatic stimuli.

Atrophy of the ligated lobe is the result of apoptosis while hypertrophy of the non-ligated lobe is due to the induction of hepatocyte proliferation. In our previous study^3^ we showed for the first time that proliferation and apoptosis can even occur in the same liver lobe. We subjected the animals to PVL of the small right lobe and to substantial PHx by removing 70% of the liver mass. In this case, the portally deprived liver lobe did not undergo atrophy but increased its size slightly. Due to the strong regenerative stimulus and despite the lack of portal venous blood supply, we observed a low rate of proliferating hepatocytes in the portally deprived lobe. We wondered now whether the net increase of liver volume was the result of inducing proliferation via the resection-induced regeneration stimulus or whether apoptosis was also affected.

Hepatic proliferation as well as apoptosis are highly dependent on sufficient energy and nutrients. Extended hepatectomy induces heavy metabolic burden which may cause mitochondrial damage. Autophagy is a highly conserved self-digestion process that enables cells to maintain hemeostasis under nutrient deprivation and other stresses^4^. The typical characteristic of autophagy is identified as engulfing damaged organelles and misfolded proteins to form autophagosomes which are subsequently degraded in lysosomes and produce new components and energy^5^. The recycling function of autophagy contributes an essential role in cellular energy balance^6^. In addition, autophagy improves hepatic metabolic homeostasis by selectively removing damaged mitochondria and maintaining functional mitochondria^7^. This arouses an interest to investigate the role of autophagy in portal vein deprived liver lobes after simultaneous PVL and PHx.

In this study we used our recently established model of PVL and PHx to study the size regulation of deportalized liver lobe and investigate the potential mechanisms.

We hypothesized that (1) there is a quantitative relationship between the final liver weight and the relative “strength” of the atrophy and regeneration stimulus.

We further hypothesized that (2) that the net change in liver volume is the result of balancing the relative” strength” of the atrophy signal and the regeneration signal as indicated by the simultaneous presence of apoptosis and proliferation markers.

In a third step, we evaluated the role of autophagy in intrahepatic size regulation.We speculated that (3) upregulation of autophagy in the ligated lobe might provide the energy needed for hepatocyte proliferation in the ligated lobe.

## Results

### Simultaneous PHx abrogated atrophy of the deportalized liver lobes in a size dependent manner

**Figure 1:**
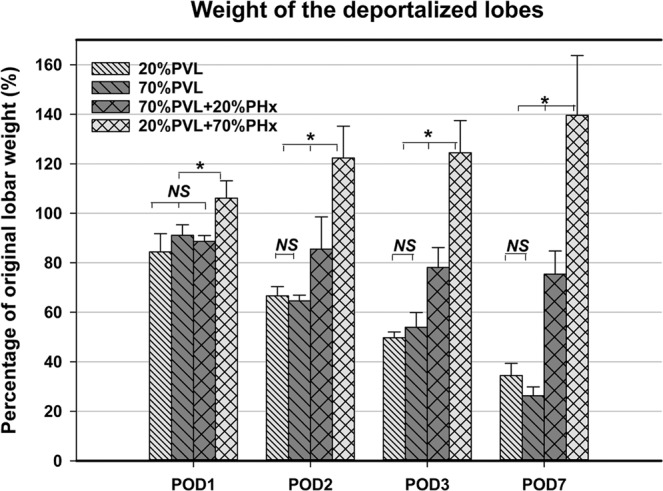
Lobar weight adjustment of the deportalized lobes without/with simultaneous PHx. The deportalized right lobe decreased in weight to similar extent after 20%PVL and 70%PVL on POD7 (33% and 26% of original lobar weight, p > 0.05). However, with a simultaneous 20%PHx the deportalized lobes decreased only to 75% of the original lobar weight (p < 0.05), whereas it increased to 140% after simultaneous 70%PHx (p < 0.05).

The weight of the ligated lobe decreased to about 1/3 after 20%PVL and 70%PVL as expected (Fig. 1). As previously reported^8^, the deportalized right lobe in 20%PVL + 70%PHx group did not decrease in weight but increased steadily. At the end of the observation period of 7 days, the deportalized lobe reached 140% of its original weight, which is almost 5-fold more than the atrophied liver lobe after 20%PVL only (p < 0.01; vs. 140% vs. 33% of original lobar weight). In contrast, after simultaneous 70%PVL + 20%PHx the deportalized lobes did undergo atrophy, but much less pronounced compared to 70%PVL only. The deportalized lobe was still 3 time larger than after 70% PVL only (p < 0.01; 75% vs. 26% of original lobar weight). Therefore, it seemed that the size of the deportalized lobes may be related to the extent of simultaneous resection: (1) without simultaneous resection leading to most pronounced atrophy; (2) with simultaneous small resection leading to a less pronounced atrophy; (3) with simultaneous large resection leading to an overall hypertrophy.

### Simultaneous PHx induced mild hepatocyte proliferation in the deportalized liver lobes

**Figure 2:**
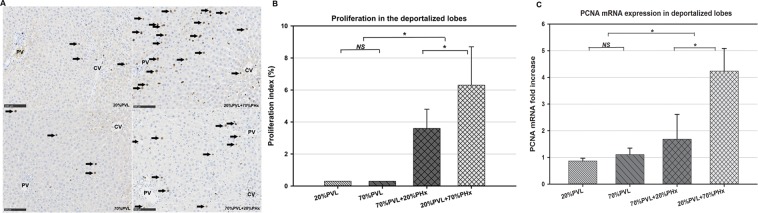
Proliferation index (PI) and BrdU-staining of the deportalized lobes without/with simultaneous PHx. (**A**) Proliferation was absent after small 20% PVL and very low after 70% (maximum PI of 0.3% on POD 2). In contrast, in both additional resection groups, the PI of deportalized lobe was more than 10-fold higher (3.6% and 6.3% on POD 2). (*p < 0.05). (**B**) BrdU-staining of deportalized lobes (RL or LLL) on POD 2. Rare proliferating cells were observed after 20%PVL and 70%PVL. In contrast, more proliferating cells were visible after simultaneous 20%PHx and 70%PHx. (Arrows indicate positive-stained cells, PV: portal vein, CV: central vein.scale marker: 100 μm, magnification: 200×). (**C)** PCNA mRNA expression in deportalized lobes after PVL only and simultaneous PHx on POD1. In deportalized liver lobe, PCNA mRNA increased more than 4 fold after simultaneous 70%PHx compared with simultaneous 20%PHx respectively 20%PVL. (*p < 0.05).

Hepatocyte proliferation in the deportalized lobes was hardly observed after PVL only, irrespectively of the extent of ligation (Fig. 2A). In contrast, after the combined procedure, we observed notable hepatocyte proliferation in the *ligated lobes* as confirmed by histological examination (Fig. 2B) and qPCR for proliferating cell nuclear antigen (PCNA) (Fig. 2C). The proliferation index (PI) of ligated lobes after simultaneous PVL and PHx both were significantly increased compared with the control groups on postoperative day (POD) 2 (p < 0.01, vs. PI = 0.3%). Interestingly, additional simultaneous 70%PHx induced significantly higher PI than additional simultaneous 20%PHx (p = 0.033, PI = 6.3% vs. 3.6%).

We then examined the gene expression of PCNA, a widely used marker of DNA replication, in deportalized lobes after simultaneous PHx and PVL only. We observed significantly higher levels of PCNA-mRNA in the deportalized lobe after simultaneous 70%PHx compared to simultaneous 20%PHx on POD 1. (*p < 0.05, fold change = 4.1 vs 1.9).

### Simultaneous PHx reduced apoptosis density remarkably in the deportalized liver lobes

**Figure 3:**
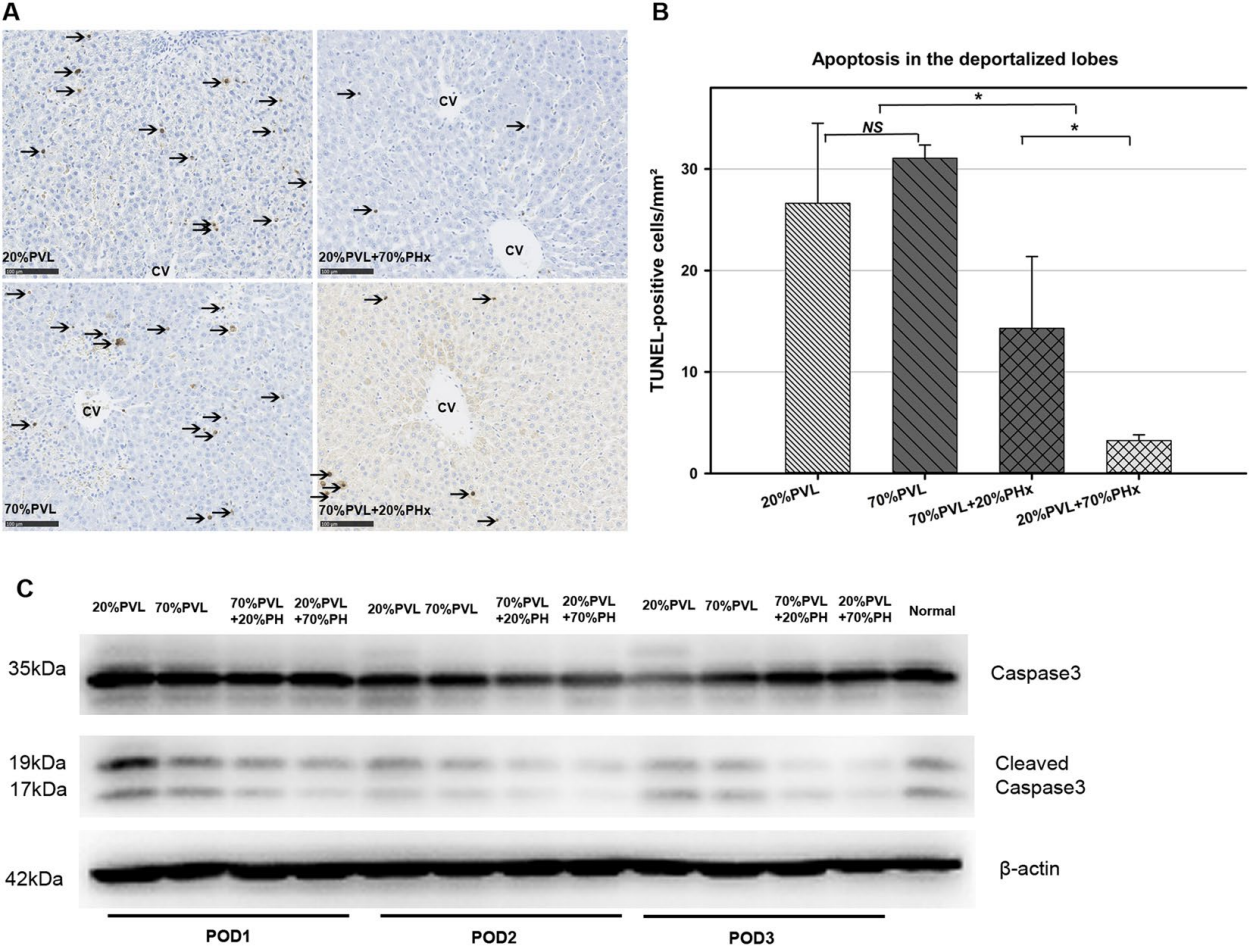
Apoptosis density of the deportalized lobes without/with simultaneous PHx on POD3. (**A**) After 20%PVL respectively 70%PVL, apoptosis density was high (26 and 31 cells/mm^2^ on POD3). In contrast, apoptosis density were significantly lower after simultaneous 20%PHx and 70%PHx (14 cells/mm^2^ and 3 cells/mm^2^, *p < 0.05). (**B**) TUNEL-staining of deportalized lobes on POD3 (upper panel: 20%PVL and 20%PVL + 70%PHx; lower panel: 70%PVL and 70%PVL + 20%PHx). After PVL only (left panel), abundant apoptotic cells were clearly visible; In contrast, only single TUNEL-positive cells were visible after simultaneous PHx (right panel) (scale marker: 100 µm, magnification: 200×).(**C**) Simultaneous PHx decreased hepatocytes apoptosis in deportalized lobe. Caspase 3/cleaved casepase 3 in deportalized lobe was assessed using Western blots. Full-length blots are presented in Supplementary Fig. 3.

Apoptosis was observed in all deportalized lobes and reached the maximum on POD3 (Fig. 3A,B). We observed a significant reduction of apoptosis in the simultaneous large resection (20%PVL + 70%PHx) compared with 20%PVL only (p < 0.01, 3 vs. 26 cells/mm^2^). At the same time, significantly less apoptotic hepatocytes were observed in the deportalized lobes after simultaneous small resection (70% PVL + 20%PHx) compared with 70%PVL only group (p < 0.01, 14 vs. 31 cells/mm^2^). Interestingly, simultaneous 70%PHx reduced apoptosis density significantly more than simultaneous 20%PHx (p < 0.01, 3 vs. 14 cell/mm^2^). In other words, the extent of resection seemed to influence apoptosis density. The larger the additional simultaneous resection, the less apoptotic cells were observed (Arrows indicate positively-stained cells, CV: central vein, scale marker: 100 μm, magnification: 200×).

Caspase 3 is an essential pro-apoptotic effector, its cleavage forms are indicator of caspase processing. Therefore western blotting for caspase 3 was performed. Protein levels of cleaved caspase 3 were markedly decreased in deportalized lobes after additional 70%PHx compared with 20%PVL and less decreased in additional 20%PHx compared with 70%PVL (Fig. 3C), both findings confirming the results obtained in the TUNEL-assay.

### Large simultaneous PHx induced more autophagy in the deportalized liver lobes than the small resection and PVL only

**Figure 4:**
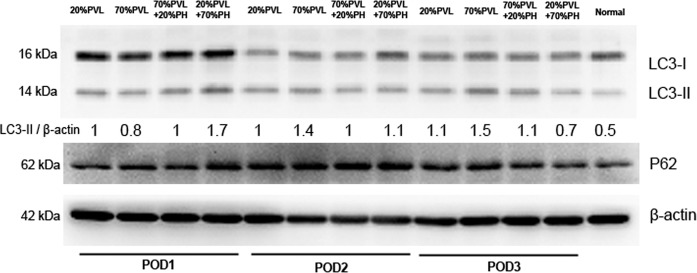
LC3 protein expression in deportalized lobe was examined with Western blots. LC3-II was elevated after simultaneous 70% PHx compared with after simultaneous 20% PHx and PVL only on POD1. Expression level of p62 was increased rapidly after simultaneous 70%PHx on POD1 and reduced rapidly on POD3. Full-length blots are presented in Supplementary Fig. 3.

After simultaneous 70%PHx or 20%PHx, autophagy-related LC3 and P62 protein levels were detected by Western Blots. The expression level of LC3-II was higher on POD1 after additional large liver resection compared with additional small liver resection and PVL only (Fig. 4), indicating an induction of autophagy. LC3-II level was elevated after 70%PVL only on POD3, showing a delayed activation of autophagy. The expression level of p62 increased rapidly on POD1 after additional large resection and reduced rapidly on POD3 compared with additional small liver resection and PVL only, indicating a rapid activation of autophagy.

## Discussion

This is the first animal model allowing to study the impact of counteracting regeneration and atrophy stimuli on the same liver lobe.

In support of our hypothesis, we observed that the net balance between the regeneration stimulus and the atrophy stimulus determined the size of the ligated liver lobe. On the *organ level*, we observed two phenomena: (I) the strong regeneration stimulus counteracting the weak atrophy stimulus caused an increase in size of the deportalized lobes; (II) in contrast, the weak regeneration stimulus counteracting the strong atrophy stimulus resulted in a lower extent of liver size decrease. On the *cellular level*, we observed in case of the size increase of the ligated lobes mild induction of proliferation and substantial reduction of apoptosis in the portally-ligated liver lobes. On the *molecular level*, we observed that the induction of hepatocyte proliferation as indicated by the increased PCNA-expression level was associated with upregulation of autophagy as indicated by the higher level of LC3II. We further observed that all three processes (hepatocyte proliferation, autophagy and apoptosis) taking place in the ligated lobe were related to the extent of additional liver resection. These observations suggest that these processes are linked and that the interplay between hepatocyte proliferation, apoptosis and autophagy is governing intrahepatic size regulation and determing the size of the ligated liver lobe.

Intrahepatic liver size regulation is gaining attention. Picard *et al*.^9^ investigated the relation between apoptosis and proliferation. They reported that suppression of hepatocyte proliferation using retrorsine in a PVL-model reduced liver regeneration of the non-ligated lobe but also reduced atrophy and reduced apoptosis in the ligated lobe as indicated by the inhibition of caspase activation. They concluded that the liver “attempt(ed) to reduce the loss of liver mass when hyperplasia of the nonligated lobes is impaired”. Zhou *et al*.^10^ observed in a rat model of 70% PHx that “the balance between hepatocyte proliferation and apoptosis is critical for liver homeostasis during liver regeneration”. In a mouse partial hepatectomy model with nuclear factor (erythroid-derived 2)-like-2 activation, liver regeneration was impaired “as a result of delayed hepatocytes proliferation and enhanced apoptosis”^11^.

A series of paper investigating the role of autophgy for liver regeneration after partial hepatectomy were published. Lin *et al*. reported that 70% liver resection in a mouse model induced autophagy. Further induction of autophagy using amiodarone enhanced liver regeneration, and reduced mortality and liver injury ^12^. Based on these observations, they concluded that autophagy plays a critical role in liver regeneration in the early phase after PHx. Toshima *et al*. also observed a protective role of autophagy in the early phase of liver regeneration after resection. They found that autophagy maintained functional mitochondria, thereby  meeting the demand for energy and supporting hepatocyte proliferation^13^. The role of autophagy in portal vein ligated liver lobe is much less elucidated. However, it is well knwon that nutrient deprivation, in other words starvation, induces autophagy^14^. Occlusion of portal vein causes lack of nutrients from gastrointestinal tract, leading to a condition of nutrient deprivation in deportalized lobe^15^.

LC3 is a well accepted marker of autophagy, which is involved in  autophagosome formation. LC3-II is the lipidation form of LC3 after cleavage, binding to the autophagosome membrane and promoting membrane elongation^16^. Therefore, the protein level of LC3-II is closely related with the amount of autophagosome. In the present experiment we also observed enhancement of liver regeneration and activation of autophagy in the deportalized lobe. Both phenomena occurred at the same time after PVL with simultaneous PHx, albeit to a different extent depending on the extent of simultaneous PHx. Specifically, the additional large PHx induced higher LC3-II expression in the deportalized lobe on POD1 than additional small PHx and PVL only. The elevation of LC3-II level was delayed after 70%PLV only.

P62 (sequestosome1/SQSTM1) is a multifunctional protein, which plays a critical role in processes such as autophagy, apoptosis, inflammation, cell survival and cell death^17^. Sha *et al*. observed in a cell culture experiment, that  p62 expression was increased rapidly before autophagy activation byar proteasome inhibition. They concluded that the increased production of p62 is essencial to sustain a high capacity for autophagy^18^. In the amimal model from Lin *et al*., the expression level of p62 was relatively increased after PHx compared with sham group^12^. In our experiment, we also observed that the expression level of p62 increased rapidly after additional large resection on POD1 and decreased also rapidly to reach basal levels on POD2.  P62 participates in the formation of autophagosomes and is consumed with cargo during the lysosome hydrolysis. The rapid degradation of p62 could indicate completion of autophagy.

Autophagy protects tissues and cells against various types of cytotoxic stress by inhibition of cellular apoptosis in different pathways^19^. Autophagy can attenuate apoptosis by inhibiting both intrinsic and extrinsic pathway of apoptosis^20^. Chow *et al*. reported that induction of autophagy reduced apoptosis via a mTOR-dependent pathway, on the other hand inhibition of autophagy increased the apoptosis activity^21^. A recent study demonstrated that autophagy played an essential role in augmenter of liver regeneration protecting cells against apoptosis^22^. In our experiment, animals subjected to simultaneous large PHx presented with higher activation of autophagy but showed significantly lower caspase activation on POD1 and POD2 compared to animals undergoing a small additional PHx.

We postulated that the additional removal of liver mass induced upregulation of autophagy in the portally deprived liver lobe which in return induced proliferation and suppressed apoptosis. The resulting size of the ligated liver lobe was related to the net balance of the counteracting stimuli which influenced the upregulation of autophagy and thereby the level of proliferation respectively apoptosis.

## Conclusion

Additional liver resection counteracts hepatic atrophy of the deportalized liver lobes by (1) inducing hepatocyte proliferation and by (2) suppressing hepatic apoptosis in a size dependent manner. The underlying mechanism might be related to a starvation-like induced activation of autophagy which in return enhances proliferation and inhibits apoptosis.

## Methods

### Animals

Animal experiments were performed in inbred male Lewis Rats (Charles River, Sulzfeld, Germany) aged 9–10 weeks (body weight 250–300 g). Rats were fed a laboratory diet with water and rat chow ad libitum and were kept under constant environmental conditions with a 12 h light–dark cycle in a conventional animal facility until harvest.

### Ethics statement

All procedures and housing of the animals were performed according to current German regulations and guidelines for animal welfare and the ARRIVE Guidelines for Reporting Animal Research^23^. The Thüringer Landesamt für Verbraucherschutz, Thuringia, Germany approved the protocols (Approval-Number: 02-024/13).

### Experimental design

**Figure 5:**
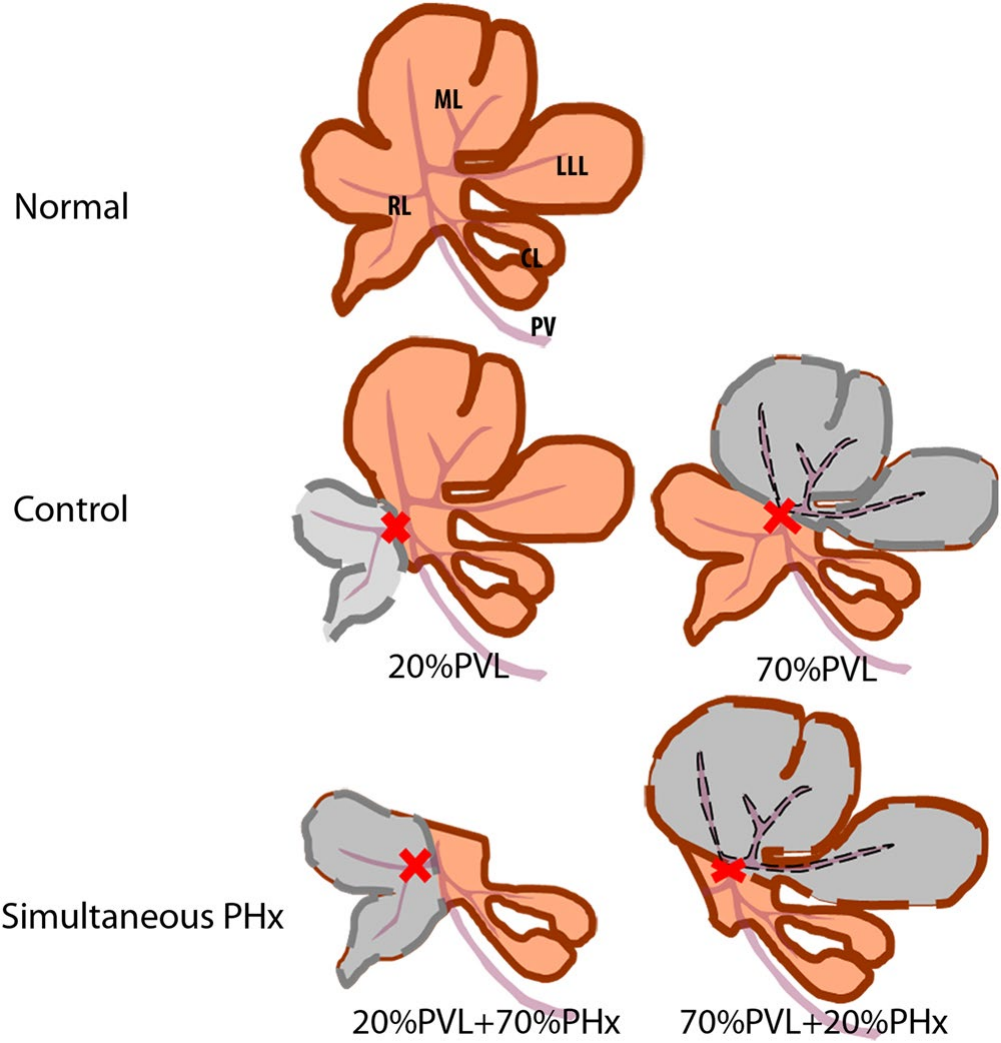
Sketches of experimental groups. (red cross: portal vein ligation; grey colored lobes: deportalized lobes; orange colored lobes: portalized lobes) RL: right lobe, ML: median lobe, LLL: left lateral lobe, CL: caudate lobe, PV: portal vein.

Male Lewis rats were allocated into two experimental groups to investigate the effect of two concurrent stimuli on intrahepatic liver lobe size regulation (Fig. 5): (i) 20%PVL + 70%PHx: ligation of right and left portal vein followed by resection of left lateral and median lobes representing removal of 70% of the liver mass (see also ref); (ii) 70%PVL + 20%PHx: ligation of right and left portal vein followed resection of right lobe representing removal of 20% of the liver mass. Control groups consisted of 20%PVL and 70%PVL to determine the relationship between the extent of ligation and atrophy. Animals in each group were harvested on postoperative day (POD) 1, 2, 3 and 7 (n = 6/group/time points).

Samples were used to investigate the releationship between liver regeneration, apoptosis and autophagy using key marker molecules such as PI and PCNA for regeneration, TUNEL and caspase3 for apoptosis and LC3B and p62 for autophagy.

### Operative procedures and postoperative management

Surgical procedures were performed under inhalation of 3% isoflurane mixed with pure oxygen at a flow rate of 0.5 L/min (isoflurane vaporizer, Sigma Delta, UK). Laparotomy was carried out via a transverse upper abdominal incision. PVL was performed carefully without injuring bile duct and hepatic artery using an operating microscope (Zeiss, magnification 10–25×, Germany). Ligation of right portal vein represented 20%PVL, while ligation of left portal vein represented 70%PVL. Additional PHx was performed using the precise vessel-oriented technique as described previously^24^. After placing a Mosquito-clamp 2–3 mm away from abdominal vena cava followed by removing the liver lobe, 2–4 piercing sutures were made to ligate each main branch of portal vein and hepatic vein prior to removing the clamp.

All animals received analgesic treatment with buprenorphine in a dose of 0.05 mg/kg body weight (Temgesic, Essex Pharma GmbH, Germany). Daily assessment of activity was carried out. Animals in each group were harvested at four observation time points (postoperative day (POD) 1, 2, 3 and 7, n = 6/group/time points). One hour before harvest, 5-bromo-2-deoxyuridine (BrdU, SIGMA-ALDRICH, St. Louis, USA) was injected via penile vein in a dose of 50 mg/kg body weight for revealing hepatocellular proliferation.

### Assessment of blood sample

Blood was collected for measurement of serum aspartate aminotransferase (AST), alanine aminotransferase (ALT), albumin and cholinesterase by using the AEROSET System (Abbott Laboratories, Wiesbaden, Germany) according to the instructions of the manufacturers.

### Liver explantation, liver weight determination

Livers were mobilized for liver explantation and photo documentation (Fig. S1). Individual liver lobes were weighed to calculate the liver weight/body weight ratio using the following formula: individual liver lobe weight (g)/body weight (g)*100%. Six additional rats were subjected to laparotomy to obtain normal values of liver weights and normal range of liver enzymes.

### Immunohistochemistry (IHC)

Liver sections of 4 µm thickness were cut after formalin fixation and paraffin embedding. BrdU-staining was performed using a monoclonal anti-BrdU antibody (Dako, Hamburg, Germany) following the protocol described previously^25^. The terminal deoxynucleotidyl transferase-mediated dUTP nick end labeling (TUNEL) staining was performed using an Apoptag peroxidase *in-situ* apoptosis-detection kit (Intergen, Purchase, NY) according to the manufacturer’s instructions.

### Assessment of proliferation and apoptosis

The analysis of proliferation index (PI) was performed with a histological image analysis tool “Histokat” (Fraunhofer MEVIS, Bremen, Germany) as described previously^8^. The result was expressed as the fraction of labeled hepatocyte nuclei to the total number of hepatocyte nuclei (accurate to 0.1%). In contrast, apoptosis density was assessed manually by counting the number of TUNEL-positive cells per observation area (magnification 400-fold) in 10 fields per animal (given as cells/mm^2^)^26^. PI was defined as high when it was more than 20%; PI was defined as moderate when it was less than 20% but more than 10%; PI was defined as low when it was less than 10%.

### Gel electrophoresis and Western blotting

Total protein was extracted from liver tissues using the RIPA buffer (sigma, R0278) and the Protease and phosphatase inhibitor cocktail (Thermo Scientific). After the concentration being quantified by BCA protein assay kit, equal amounts of protein from animals of the same group were pooled. Western blotting was performed as described previously^27^. Rabbit anti-light chain 3 (LC3B; 1:1000, Abcam), rabbit anti-Caspase3 (1:1000, Cell signaling Technology), rabbit anti-SQSTM1/p62 (8025, 1:1000, Cell signaling Technology) and rabbit anti-glyceraldehyde-3-phosphate dehydrogenase (GAPDH; 1:10,000, Cell signaling Technology) were used as primary antibodies. Goat polyclonal antibody to rabbit IgG (1:5000) was used as secondary antibody. The membranes were probed using enhanced chemiluminescence western blotting substrate (GE Healthcare) and exposed to high sensitivity film (GE Healthcare) or digitalized with Fusion FX7 (Labtech International Ltd, Heathfield, United Kingdom). The signal intensity of each protein band was quantified using Gel Analyzer Module provided by ImageJ (National Institutes of Health, Bethesda, MD). All Western blots were repeated 3 times.

### Quantitative real-time PCR analysis

Total RNA was isolated from the liver tissues using the RNeasy kit (Qiagen, Hilden, Germany), and the procedure was performed according to the manufacture’s instruction. The concentration of RNA was quantified by Nano-Drop spectrophotometer (ND-100, PEQLAB, Erlangen, Germany). Complementary DNA was reverse transcribed from total RNA (2–5 μg) using the First-Strand cDNA synthesis KIT (Invitrogen, Carlsbad, USA). Two steps real-time PCR was performed in an M3000P QPCR System (Stratagene, La Jolla, USA), using the Brilliant probe-based QPCR Mater Mix kit (Agilent, Santa Clara, USA). The sequences of primers (eurofins Genomics, Germany) and probes (Universal Probe Library) were as follows: proliferating cell nuclear antigen (PCNA): TGAACTTTTTCACAAAAGCCACT, TGTCCCATGTCAGCAATTTTA, and probe #94; hypoxanthine guanine phosphoribosyltransferase (HPRT): GACCGGTTCTGTCATGT CG, ACCTGGTTCATCATCACTAATCAC, and probe #95. Relative quantification gene expression of fold change was calculated by using HRPT as an endogenous control. Quantitative real-time PCR was performed for three times.

### Statistical analysis

The data, expressed as mean ± standard deviation, were analyzed using SigmaPlot 13.0 (Statcon, Witzenhausen, Germany). Differences between paired groups were analyzed using the two-tailed paired samples Student’s t-test and multiple groups were compared using the one way independent ANOVA test. Differences were considered statistically significant if p-values were less than 0.05.

## Supplementary information

Supporting information.

## Data Availability

All data generated or analyzed during this study are included in this presented article (and its Supplementary Files). Parts of the data in this manuscript were presented in Dr. Wei’s thesis. (https://www.db-thueringen.de/servlets/MCRFileNodeServlet/dbt_derivate_00039437/disswei.pdf).
