# Supplementary material for: Size of portally deprived liver lobe after portal vein ligation and additional partial hepatectomy: Result of balancing proliferation and apoptosis
Source: Sci Rep. 2020 Mar 17;10:4893. doi: 10.1038/s41598-020-60310-0 (PMC7078252; doi:10.1038/s41598-020-60310-0)
Supplement: Supplementary file 1 — Supporting information. [file 41598_2020_60310_MOESM1_ESM.docx]

**Size of portally deprived liver lobe after portal vein ligation and additional partial hepatectomy: Result of balancing proliferation and apoptosis**

Weiwei Wei, *MD* ^a^* Chuanfeng Hua, *MS* ^a^* Tianjiao Zhang, *MD* ^b^  Olaf Dirsch, *PhD* ^c^

Felix Gremse, *PhD* ^d^ André Homeyer, *PhD* ^e^ Utz Settmacher, *MD* ^a^ Uta Dahmen, *MD* ^a^**

^a^ Department of General, Visceral and Vascular Surgery, Jena University Hospital, Jena, Germany

^b^ Department of Radiotherapy and Radiooncology, Jena University Hospital, Jena, Germany

^c^ Institute of Pathology, Klinikum Chemnitz gGmbH, Chemnitz, Germany

^d^ Department of Experimental Molecular Imaging, RWTH Aachen University, Aachen, Germany

^e^ Fraunhofer Institute for Medical Image Computing MEVIS, Bremen, Germany

* These authors contributed equally to this work.

**Corresponding author

E-mail: [Uta.Dahmen@med.uni-jena.de](mailto:Uta.Dahmen@med.uni-jena.de) Tel.: 03641-9325350 Fax: 03641-9325352

Supporting information

Surgical procedures were well tolerated. As expected, the severity of surgical stress as indicated by the hepatic enzyme release was dependent on the extent of PHx respectively PVL. After all procedures, maximal hepatic damage occurred on POD 1 and recovered fully within 7 days as indicated by liver enzymes (ALT, AST) (Table 1). However, we did not see any effect of PHx/PVL on hepatic synthetic function during the observation as indicated by serum levels of albumin and cholinesterase (data not shown).

**Table1. Level of surgical stress as indicated by the release of transaminases**

| Groups | Surgery | ALT (IU/L) | | | | AST (IU/L) | | | |
| --- | --- | --- | --- | --- | --- | --- | --- | --- | --- |
|  |  | **POD1** | **POD2** | **POD3** | **POD7** | **POD1** | **POD2** | **POD3** | **POD7** |
| PVL only | 20%PVL | 106.8 | 68.8 | 52.0 | 42.8 | 429.4 | 129.4 | 100.6 | 90.6 |
|  | 70%PVL | 611.6 | 499.6 | 243.0 | 62.2 | 823.8 | 609.6 | 300.5 | 93.2 |
| Simultaneous | 70%PVL+ 20%PHx | 934.6 | 522.9 | 123.1 | 49.2 | 1505.9 | 580.6 | 167.2 | 77.6 |
| PVL+PHx | 20%PVL+ 70%PHx | 1383.4 | 334.0 | 112.6 | 40.1 | 1723.7 | 495.0 | 192.6 | 83.0 |

The level of transaminases was related to the extent of liver mass subjected to ligation or resection, which reflected the liver injuries as well. All values were expressed as mean value. AST: alanine aminotransferase; AST: aspartate aminotransferase; POD: postoperative day.


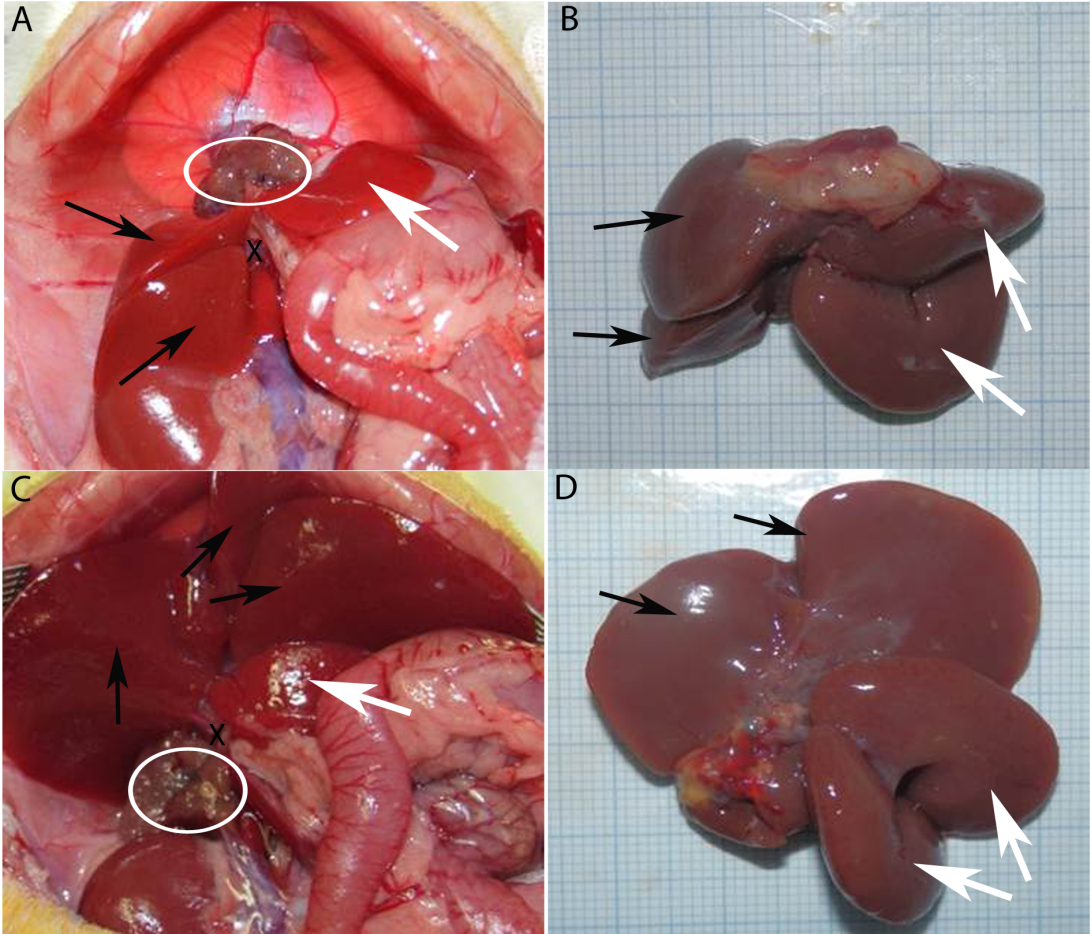


**Supplementary Figure 1. Intraoperative images after combined portal vein ligation (PVL) and partial hepatectomy (PHx)** (**A, C**) **and images of explanted livers on POD 7 to illustrate liver lobe size regulation** (**B, D**). (A) Intraoperative situs after 20%PVL+70%PHx: depicting portally deprived right lobes (black arrows) with darker red color after PVL (black cross) and stumps of left lateral and median lobes (white circle) after resection, and remnant caudate lobes with fresh red color (white arrow); (B) Explanted liver after 20%PVL+70%PH on POD7: demonstrating the slight hypertrophy of the deportalized right lobe (black arrows) and the substantial hypertrophy of the regenerating caudate lobes (white arrows); (C) Intraoperative situs after 70%PVL+20%PHx: depicting portally deprived left lateral and median lobes (black arrows) with darker red color after PVL (black cross) and stumps of right lobes (white circle), and remnant caudate lobes with fresh red color (white arrow); (D) Explanted liver after 70%PVL+20%PHx on POD7: demonstrating discrete atrophy of left lateral and median lobes (black arrows) and the substantial 4-fold increase in size of the regenerating caudate lobes (white arrows).

**
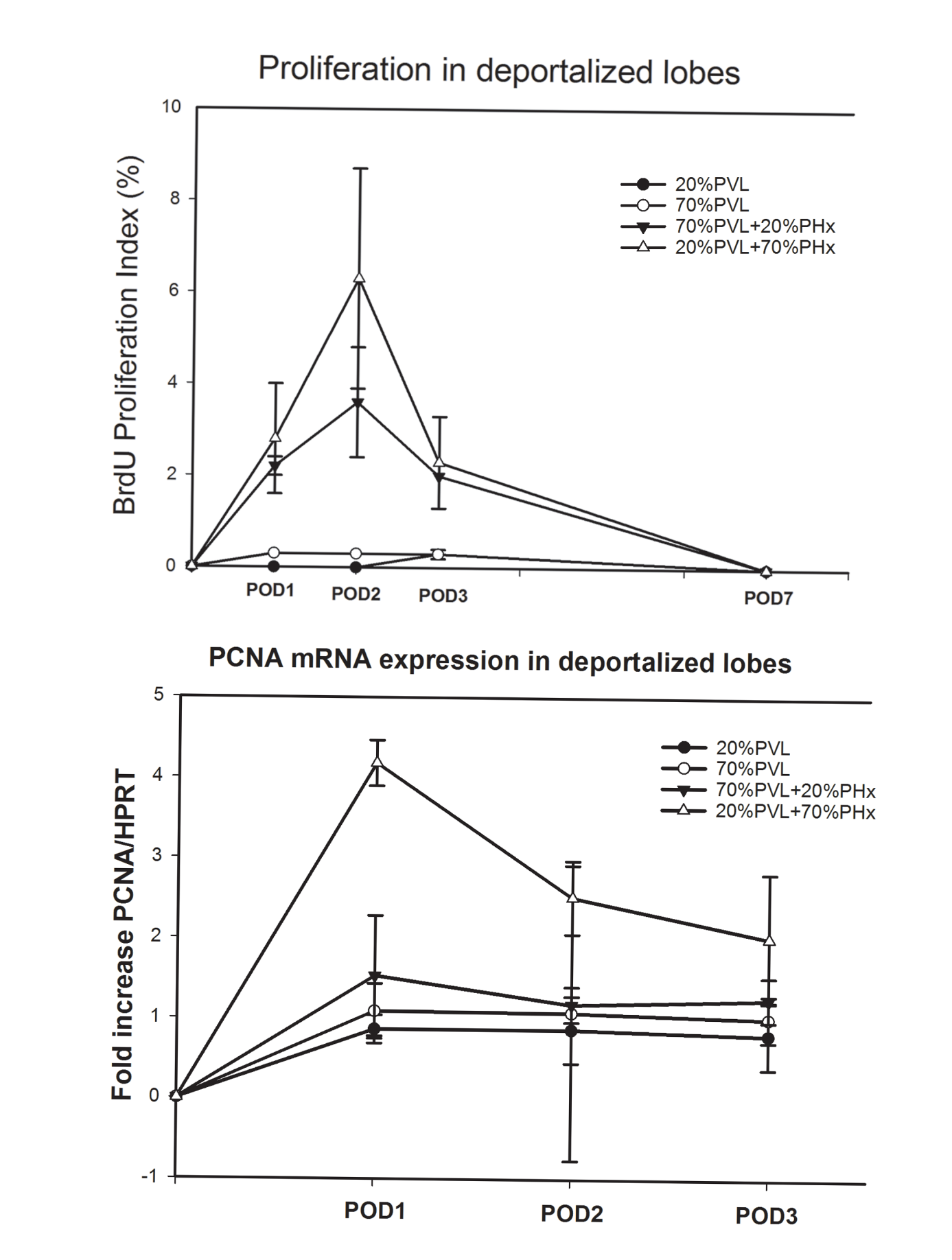
**

**Supplementary Figure 2. Proliferation index (PI) and PCNA mRNA expression level**

**
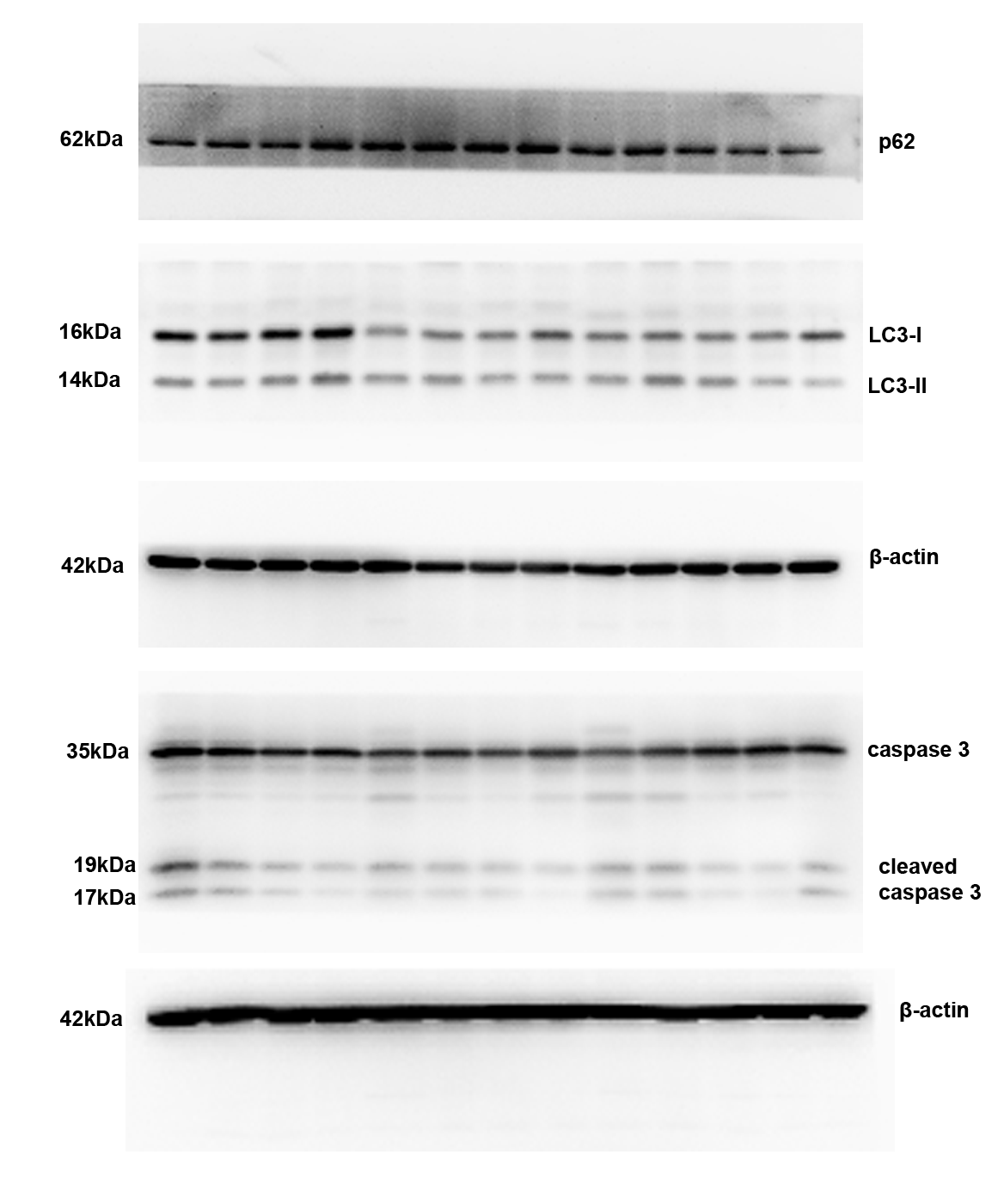
**

**Supplementary Figure 3. Full length western blots (POD1-POD3)**

**
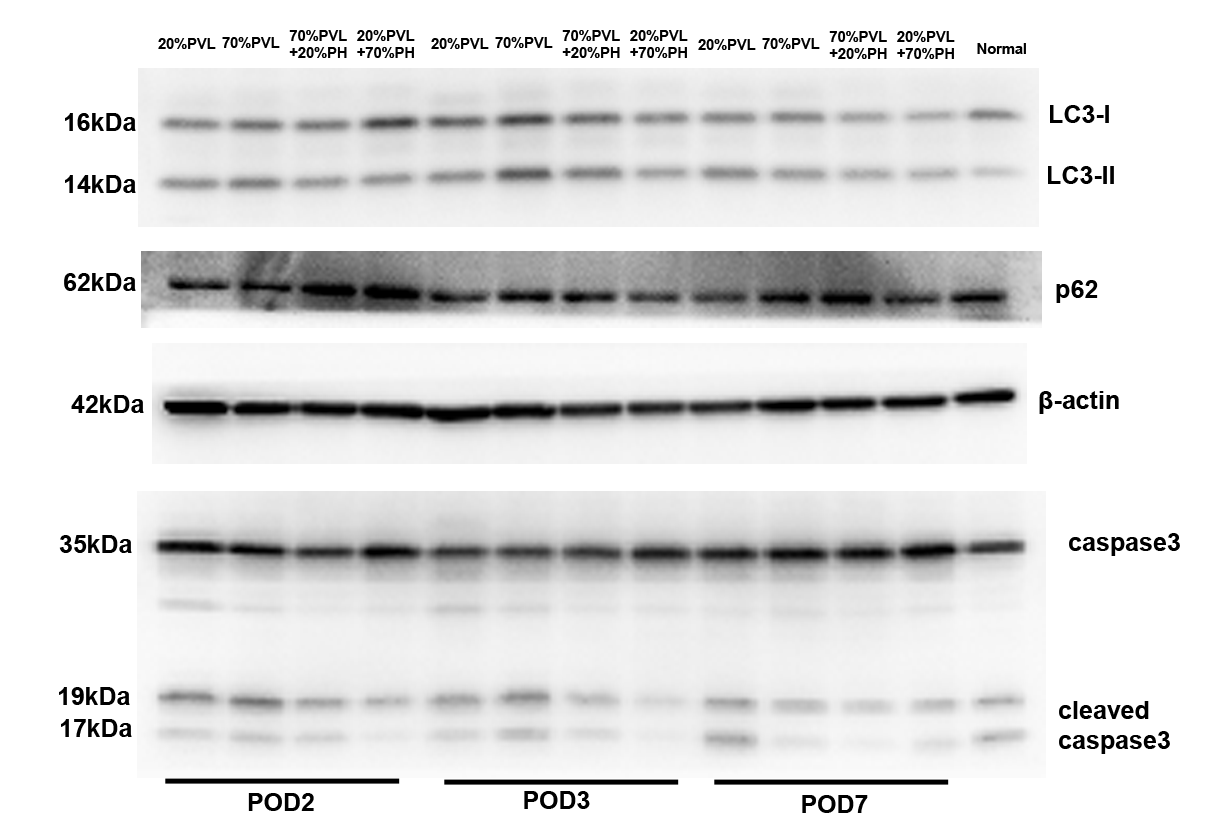
**

**Supplementary Figure 4. Western blots included POD7**
